# Supplementary material for: Physiological Changes in Chicken Embryos Inoculated with Drugs and Viruses Highlight the Need for More Standardization of this Animal Model
Source: Animals (Basel). 2022 Apr 29;12(9):1156. doi: 10.3390/ani12091156 (PMC9099557; doi:10.3390/ani12091156)
Supplement: Supplementary file 1 [file animals-12-01156-s001.zip › animals-1644052-supplementary.pdf]

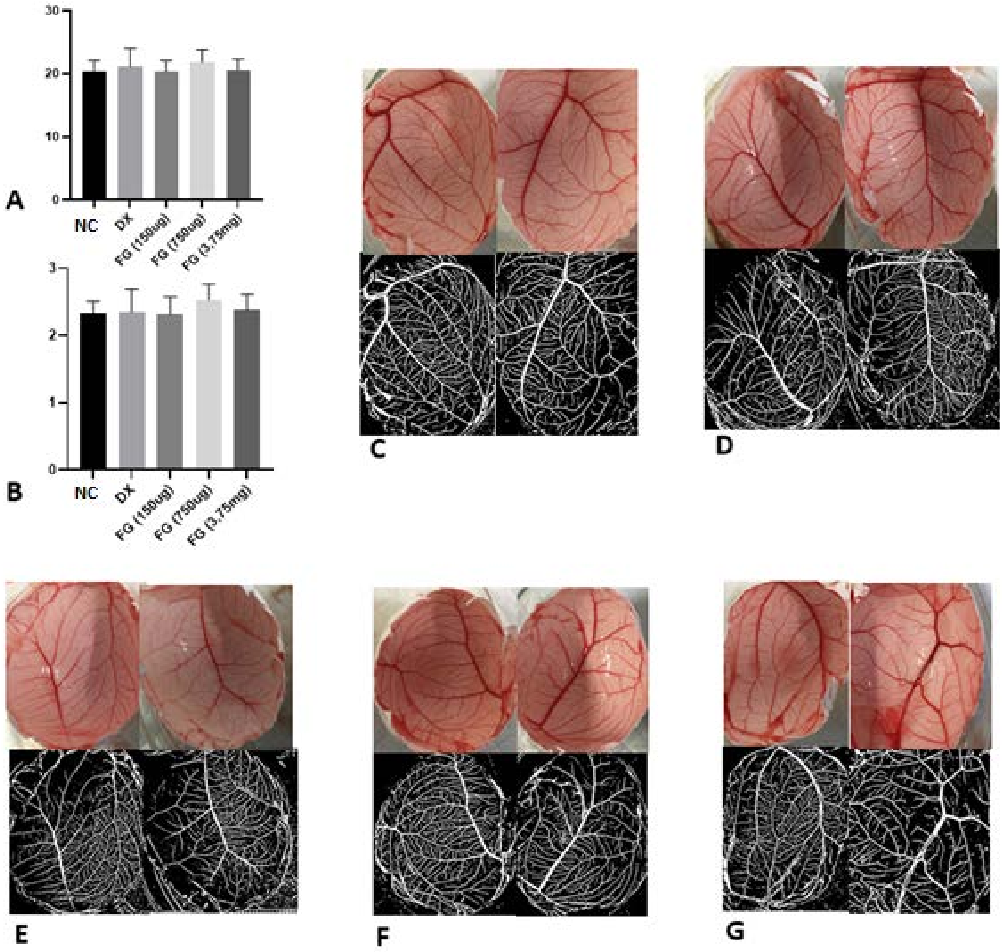

Figure S1. Angiogenesis was measured by vascular density and vessel length density using the software ImageJ with the plugin Vessels Analysis 58. The CE was inoculated at 3 EID and evaluated at 12 EID. (**A**) vascular density, (**B**) vessel length density, (**C**) Photos CAM (NC), (**D**) Photo CAM treated with DX, (**E**) Photo CAM treated with FG (150 µg/CE), (**F**) Photo CAM treated with FG (750µg/CE), (**G**) Photo CAM treated with FG (3.75 mg/CE).

Table S1. Maximum and minimum values and median of hematological analysis from CE infected with *Gammacoronavirus* and NC.

	**Virus**			**NC**		
	**Minim**	**Maxim**	**Median**	**Minim**	**Maxim**	**Median**
Hc	13	18	15	19	23	21
Hb 1/3Hc	4.33	6.00	5.00	6.33	7.67	7.00
Hb Cyanmetemoglobin	5.02	7.39	6.60	7.70	8.23	8.09
Erithrocytes × 10^6	1.22	1.79	1.68	1.90	2.24	2.02
MCV	80.81	121.10	92.99	83.33	147.50	87.21
MCH	32.37	42.74	37.70	29.88	58.28	38.40
MCHC	35.00	47.06	39.53	35.86	44.13	39.60
Leukocytes	3500	54,000	6000	5500	11,000	7750
Heterophils	3325	45,360	5640	5280	10,340	6788
Limphocytes	70	6480	180	120	1710	385
Monocytes	0	1080	70	0	110	0
Eosinophils	0	0	0	0	0	0
Thrombocytes	9000	51,500	19,000	18,000	29,500	20,500

Table S2. Maximum and minimum values and median of metabolites and minerals from the serum and AF of CE from CE infected with *Gammacoronavirus* and NC.

		Serum		Allantoic	
		**Virus**	**NC**	**Virus**	**NC**
UA	Minimum	4.24	1.27	7.15	1.27
	Maximum	36.94	50.07	85.39	50.07
	Median	24.67	17.85	56.93	11.39
Creat	Minimum	0.19	0.26	0.33	1.63
	Maximum	0.96	2.94	2.75	4.92
	Median	0.435	1.235	1.05	3.155
ALP	Minimum	235.7	736.5	18.2	58.2
	Maximum	3845	3388	123.1	82.9
	Median	2476	1651	24.1	68.1
GGT	Minimum	10.2	77.4	40	58
	Maximum	136	356	434	346
	Median	89.6	288	183	166
ALT	Minimum	24	40	20	20
	Maximum	100	220	120	80
	Median	53	54	60	40
AST	Minimum	54	120	120	220
	Maximum	560	780	380	920
	Median	221	452	150	640
Ca	Minimum	2.5	20.5	4.4	10.4
	Maximum	21.5	146.4	39	34.2
	Median	8.45	118.8	14.2	15.8
P	Minimum	2.7	1.4	2.5	11.6
	Maximum	8.4	9.7	18.3	29.4
	Median	5.45	8.1	8.7	17.7
CRP	Minimum	10.8	15	26	40
	Maximum	35.4	38	100	152
	Median	26	23	56	86

Table S3. Maximum and minimum values and median of hematological analysis from CE treated with DX at 10 EID and their respective NC.

	NC			DX		
	**Minimum**	**Maximum**	**Median**	**Minimum**	**Maximum**	**Median**
Hc	17.0	22.0	20.5	13.0	21.0	17.5
Hb 1/3Hc	5.67	7.33	6.84	4.33	7.00	5.84
Hb Drabkin	6.48	8.75	7.86	5.16	8.75	7.38
Erithrocytes	1,730,000	2,530,000	2,012,500	1,205,000	1,940,000	1,537,500
MCV	86.96	112.50	98.78	95.74	135.30	108.10
MCH	34.58	40.91		40.98	64.00	
MCHC	35.00	41.63	39.04	37.56	47.45	42.71
Leukocytes	1000	5000	4000	500	7500	2500
Heterophils	980	4500	3600	495	6300	2300
Limphocytes	20	810	210	5	1200	200
Monocytes	0	0	0	0	0	0
Eosinophils	0	45	0	0	210	0
Thrombocytes	11,500	22,500	14,000	1000	15,500	8500

Table S4. Maximum and minimum values and median of hematological analysis from CE treated with FG at 10 EID and their respective NC.

	NC			FG		
	**Minimum**	**Maximum**	**Median**	**Minimum**	**Maximum**	**Median**
Hc	19.0	28.0	21.0	14.0	25.0	17.0
Hb 1/3Hc	6.33	9.33	7.00	4.67	8.33	5.67
Hb Drabkin	7.08	9.17	8.09	5.58	9.76	7.76
Erithrocytes	1,125,000	2,600,000	1,772,500	1,135,000	1,995,000	1,760,000

MCV	92.39	125.30	104.20	76.92	195.60	138.80
MCH	31.24	75.29	48.04	36.19	50.04	44.19
MCHC	32.75	42.70	37.63	38.15	49.21	39.63
Leukocytes	2000	8500	3000	500	8500	3250
Heterophils	1780	8160	2880	475	7735	3218
Limphocytes	60	340	120	25	640	150
Monocytes	0	0	0	0	85	0
Eosinophils	0	0	0	0	0	0
Thrombocytes	5750	34,000	14,250	2500	39,500	7000

Table S5. Maximum and minimum values and median of metabolites and minerals from the serum and AF of CE treated with FG and DX inoculated at 10 EID and their respective NC.

		Serum		Allantoic		Serum		Allantoic	
		NC	DX	NC	DX	NC	FG	NC	FG
UA	Minim	1.83	0.63	17.52	0.76	2.53	2.64	33.65	17.46
	Maxim	32.13	22.8	115.4	129.9	22.15	38.71	127.8	174.7
	Median	10.82	12.21	53.24	76.43	9.835	7.98	109.4	82.23
Creat	Minim	0.15	0.12	2.7	1.1	0.41	0.28	1.81	2.16
	Maxim	1.08	3.06	4.26	5.67	2.34	1.98	4.29	4.65
	Median	0.51	0.695	3.735	2.18	1.14	0.51	2.16	3.01
ALP	Minim	562.6	904.1	9.1	21.5	1114	1830	8.5	9.8
	Maxim	4157	17399	130.9	150.8	3110	2661	98	126.3
	Median	2460	2263	31.9	75	1404	2473	39.1	35.8
GGT	Minim	5.1	2.4	1.3	4.3	3.1	1.1	7.4	4.7
	Maxim	229	232	27	31	228	204	22.9	21
	Median	14.4	16.4	8.35	17.8	33.3	28	13.95	12.9
ALT	Minim	14.8	25.8	6	2	5.6	6.2	2	2
	Maxim	437.9	428	10	21	118	364	15	29
	Median	71.4	123	8	14.5	41.95	145.8	8	10
AST	Minim	11.7	39	5.9	6	15	39.8	3.2	4
	Maxim	63	200.4	31	20.8	74.4	71.5	31.1	17.9
	Median	38.8	79.2	16	14	61.8	42.7	12.6	6
Ca	Minim	3.1	5.6	3.4	0.8	2.6	3.6	6.3	2.6
	Maxim	30.6	94.2	10.2	41.6	40	60	25.3	14.7
	Median	5.3	20.9	5.8	12.1	16.9	9.8	10.04	6.3
P	Minim	1.79	0.28	15.6	16.1	2.34	2	4.7	11.1
	Maxim	10.8	13.2	32.5	47.6	15.8	9	29.5	37.2
	Median	3.94	4.03	23.4	20.8	7.8	4.525	15.4	22

Table S6. Maximum and minimum values and median of metabolites and minerals from the serum of CE treated with FG and DX inoculated at 12 EID and NC.

		NC	FG	DX
UA	Minimum	5.95	5.72	3.42
	Maximum	10.5	30.18	28.8
	Median	8.7	7.93	5.34
Creat	Minimum	0.32	0.19	0.71
	Maximum	2.76	1.3	1.92
	Median	1.62	0.42	1.47
ALP	Minimum	1754	2071	1528
	Maximum	2566	2980	2391
	Median	2200	2180	1896
GGT	Minimum	9.6	5.6	2.7
	Maximum	104.4	164	108
	Median	37.5	40.2	82.8
ALT	Minimum	4.3	10.6	49.4
	Maximum	150	534	95.4

	Median	58.2	22.5	75
	Minimum	57.9	65.9	124.3
AST	Maximum	259.2	175.8	1319
	Median	112.2	104.4	390.3
	Minimum	9.7	4.1	5.9
Ca	Maximum	32.4	31.5	28.8
	Median	13.8	21.3	9.9
	Minimum	1.1	1.5	5.9
P	Maximum	11.4	9	13.2
	Median	4.4	3.8	9.5

Table S7. Maximum and minimum values and median of hematological analysis from CE treated with FG and DX at 12 EID and NC.

	NC			FG			DX		
	**Minim**	**Maxim**	**Median**	**Minim**	**Maxim**	**Median**	**Minim**	**Maxim**	**Median**
Hc	23	31	30	24	34	29	18	26	26
Hb 1/3Hc	7.67	10.33	10.00	8.00	11.33	9.67	6.67	8.67	8.67
Hb Drabkin	8.00	14.08	9.34	8.58	12.13	10.62	6.90	9.76	7.84
Erithrocytes × 10^6	1.90	3.21	2.55	2.02	3.02	2.74	1.60	2.29	1.86
MCV	80.99	131.60	117.70	89.66	118.80	112.50	113.00	139.80	125.80
MCH	28.23	53.32	42.11	36.07	42.45	39.74	39.37	42.62	41.33
MCHC	31.14	45.43	34.86	31.14	43.58	35.55	30.17	37.54	32.87
Leukocytes	2500	5000	4250	2500	8000	4500	6000	30,000	14,000
Heterophils	2175	4500	3725	1800	7280	3893	5820	29,700	15,593
Limphocytes	325.0	650.0	427.5	315	720	571	140	500	220
Monocytes	0.0	100.0	47.5	0	100	75	0	0	0
Eosinophils	0	0	0	0	0	0	0	0	0
Thrombocytes	4000	7500	5000	4000	7000	5750	6000	23,500	15,000
